# Lineage Relationship of Direct-Developing Melanocytes and Melanocyte Stem Cells in the Zebrafish

**DOI:** 10.1371/journal.pone.0021010

**Published:** 2011-06-16

**Authors:** Robert C. Tryon, Charles W. Higdon, Stephen L. Johnson

**Affiliations:** Department of Genetics, Washington University School of Medicine, St. Louis, Missouri, United States of America; Texas A&M University, United States of America

## Abstract

Previous research in zebrafish has demonstrated that embryonic and larval regeneration melanocytes are derived from separate lineages. The embryonic melanocytes that establish the larval pigment pattern do not require regulative melanocyte stem cell (MSC) precursors, and are termed direct-developing melanocytes. In contrast, the larval regeneration melanocytes that restore the pigment pattern after ablation develop from MSC precursors. Here, we explore whether embryonic melanocytes and MSCs share bipotent progenitors. Furthermore, we explore when fate segregation of embryonic melanocytes and MSCs occurs in zebrafish development. In order to achieve this, we develop and apply a novel lineage tracing method. We first demonstrate that Tol2-mediated genomic integration of reporter constructs from plasmids injected at the 1–2 cell stage occurs most frequently after the midblastula transition but prior to shield stage, between 3 and 6 hours post-fertilization. This previously uncharacterized timing of Tol2-mediated genomic integration establishes Tol2-mediated transposition as a means for conducting lineage tracing in zebrafish. Combining the Tol2-mediated lineage tracing strategy with a melanocyte regeneration assay previously developed in our lab, we find that embryonic melanocytes and larval regeneration melanocytes are derived from progenitors that contribute to both lineages. We estimate 50–60 such bipotent melanogenic progenitors to be present in the shield-stage embryo. Furthermore, our examination of direct-developing and MSC-restricted lineages suggests that these are segregated from bipotent precursors after the shield stage, but prior to the end of convergence and extension. Following this early fate segregation, we estimate approximately 100 embryonic melanocyte and 90 MSC-restricted lineages are generated to establish or regenerate the zebrafish larval pigment pattern, respectively. Thus, the dual strategies of direct-development and MSC-derived development are established in the early gastrula, via fate segregation of the two lineages.

## Introduction

In early development the embryo must faithfully establish lineages capable of generating all essential tissue types. In zebrafish, by 1 day post fertilization (dpf) many of these lineages have begun differentiation and the basic vertebrate body plan is clearly distinguishable. Part of this process of segregating lineages during early development is the establishment of adult stem cells [1], [2]. While these stem cells are primarily used for generating the large numbers of cells required in the adult following metamorphosis, they are also used in later larval development for quality regulation or regenerating damaged tissue [2], [3].

The relationship between early, direct-developing lineages responsible for the primary differentiated tissue of the embryo and the adult stem cell that regulates later development and homeostasis is unknown for most cell types. Two examples of tissues that are initially and temporarily established in the embryo and then replaced from an adult stem cell pool are the primitive and definitive blood lineages [4], [5] and the direct-developing and stem cell-derived melanocyte lineages [1], [2], [6]. Different embryonic origins for primitive and definitive blood lineages is suggested by their different sites of development; whereby the primitive blood first arises in the intermediate cell mass [7], [8] and rostral blood island [9], the definitive lineages are first evident in the posterior blood island [4], and later the bone marrow (in mammals), or the kidney (in fishes).

In contrast, all pigment cells (with the exception of the retinal-pigmented epithelium, or RPE), including the embryonic and adult melanocytes are derived from the neural crest [10]–[13]. The neural crest is a pluripotent population of cells specific to vertebrates, which generates a diverse variety of cell fates, including neurons, glia, facial cartilage and bone, and pigment cells [14]. In addition to generating the cells that develop during embryonic stages, the neural crest also sets aside stem cells for larval repair and adult development [1]–[3], [15], [16]. How cells of different fates are lineage related is the subject of intense investigation [17]–[22] but how cells of different developmental strategies (direct development vs. stem cell-based development) are related remains largely unexplored.

The zebrafish pigmentary system provides one useful means of studying direct-developing and stem cell-derived lineages. First, several studies show that the embryonic melanocyte develops directly, without a renewing stem cell intermediate prior to 3 dpf, while most post-embryonic melanocytes (after 3 dpf) develop from an *erbb3b*-dependent melanocyte stem cell (MSC) [1]–[3], [23]. Although no markers for the zebrafish MSC have been identified, their presence is revealed by regeneration of melanocytes following laser ablation [24] or treatment with melanocytotoxic prodrugs such as 4-hydroxyanisole (4-HA) or (2-morpholinobutyl)-4-thiophenol (MoTP) [25].

To further explore the relationship between direct-developing melanocyte lineages and MSC lineages in the zebrafish larvae, we applied a novel clonal analysis method. First, we developed a Tol2 transposon bearing a Xenopus *ef1α* promoter driving GFP (Xef1α>GFP) as a reporter for integration. We find that when injected at the 1–2 cell stage, the transposon integrates at a range of stages, centered at the dome stage (∼4.3 hpf), making its use suitable for a wide variety of clonal analyses. To assess whether direct-developing melanocytes and MSCs share a common progenitor, we used a transposon bearing a melanocyte-specific expression reporter, the fugu *tyrosinase related protein 1* promoter driving GFP (f*Tyrp1*>GFP) [26]. These experiments show that most melanocyte-labeling integrations occur in precursors giving rise to both direct-developing and stem cell-derived lineages, while a minority of integrations only label direct-developing lineages or stem cell-derived lineages. Analysis of clone size and clone dispersion along the anterior-posterior axis suggests that integration in the restricted lineages occurs after segregation of direct-developing and MSC fates from a neural crest cell progenitor. From our results we estimate the embryo has approximately 50–60 melanocyte-producing neural crest progenitors prior to segregation, and after fate segregation the embryo has approximately 100 direct developing melanocyte lineages and 90 lineages that generate MSCs during the period of transposon integration.

## Results

### Time of integration of Tol2 transposon following 1–2 cell stage injections

**Figure 1:**
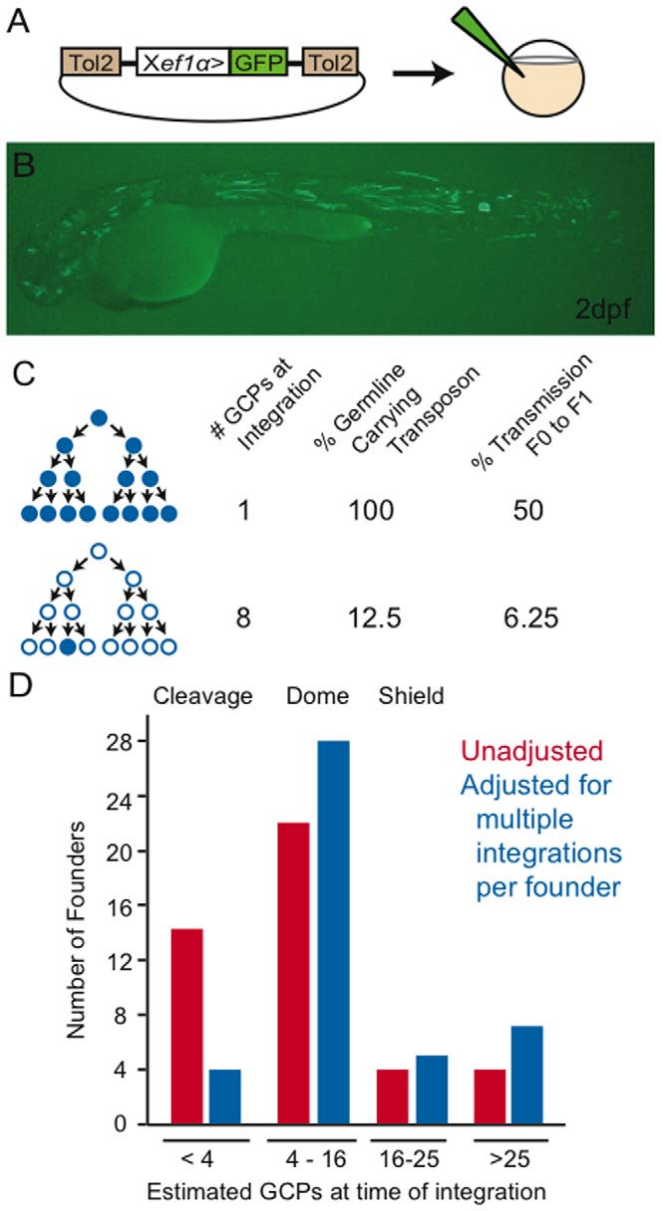
Determining integration time of the Tol2 transposon following injection in 1–2 cell embryos. (A) A plasmid construct containing the ubiquitously expressed Xenopus *ef1α* promoter driving GFP (Xef1α>GFP) and flanked by Tol2 transposon arms was co-injected with transposase mRNA into 1–2 cell embryos. (B) Following injection, typical mosaic Xef1α>GFP expression at 2 days. Note that a small minority of cells, in a wide variety of tissues, is labeled throughout the embryo. (C) Model relating the size of the germ cell precursor (GCP) population at the time of integration of the Xef1α>GFP transposon to transmission rates from F0 founders to their F1 progeny. (D) F0 founders grouped by the developmental stages at which integration took place, as inferred from the number of calculated GCPs. Number of GCPs based on previous counts of *vasa* mRNA expressing cells at various stages of development [30], [31]. Note that the majority of integration events occur after the mid blastula transition and prior to the end of the shield stage (59% unadjusted, 75% adjusted).

We were first interested in whether we could use Tol2 transposon integration following injection at the 1–2 cell stage for generating sufficiently small clones for clonal analysis of melanocyte development. We chose not to pursue clone generation from later stage injections, as previous work in our lab generating Tol2-based enhancer trap lines showed that Tol2 integration and transmission to the germline was rare following injection at the 4 or 8 cell stage (McMahon, R and Johnson, S.L., not shown). Moreover, injection of a transposon bearing the Xef1α>GFP reporter (Figure 1A) at the 1–2 cell stage typically resulted in highly mosaic embryos with only a small minority of labeled cells (Figure 1B).

To assess the timing of integration, we co-injected the Xef1α>GFP transposon with transposase mRNA into wt embryos, selected for embryos that showed GFP expression, reared them to maturity, and bred males to reveal whether they transmitted the transposon to their progeny. We reasoned that we could use the fraction of labeled F1 progeny from transmitting F0 males to estimate the time of initial transposon integration. To simplify this inference we are assuming all integrations occur in the G1 phase of the cell cycle, prior to duplication of the genome. A single integration of the transposon into the cell that gives rise to the entire germline lineage (1-cell embryo) would result in the entire germline carrying the transmissible marker. Since gametes are haploid derivatives of a diploid genome, ½ of the offspring would inherit the transposon (Fig. 1C). Alternatively, a single integration at a later stage of development when the population of germ cell precursors (GCPs) has expanded will result in a smaller proportion of offspring inheriting the transposon.

The number of germ line precursors in the zebrafish at different stages has been determined by *vasa* mRNA localization [27], [28]. Those studies show that each cell of the zygote, 2-cell and 4-cell stage embryo stains for *vasa*. The number of *vasa* expressing cells remains 4 between the 4-cell and 1000-cell stage, as somatic fates are segregated from the GCPs at each cell division. The germline quits segregating somatic fates at the midblastula transition (1000 cells) and subsequent cell divisions expand the GCP population to between 4 and 16 cells by the 4,000-cell dome stage. Shield stage embryos show *vasa* mRNA expression in 16–25 cells, with limited additional expansion of the cell population by 24 hpf (between 25 and 50 GCPs depending on the genetic background). Consequently, the number of germ cell precursors present at the time of integration can be readily translated to early developmental stages using previously reported GCP counts [27], [28].

Our analysis of transmission from 44 males showed labeled progeny proportions ranging from 0.4% to 27.1% (Table 1). Converting these percentages to the number of GCPs present at the time of integration allows us to infer when transposon integration occurs using previously reported GCP population size at different developmental stages. If we assume no polyclonal labeling we find the median number of GCPs at the time of integration is 6.9 (Table 1), or during dome stage. We note that this analysis is biased in at least two ways. First, very late integrations would tend to result in very small clones, that might not be identified in limited progeny testing (we tended to screen between 100 and 250 embryos from each male, before accepting a negative result). Second, the relatively large fraction of transmitting males (44%; 39/89 in one subset of tested fish), suggests that a portion of the F0 founders analyzed likely had multiple germline integrations, which would tend to make clone sizes appear larger, and erroneously suggest earlier integration times. Assuming independence of integration events then allows us to consider a binomial distribution of monoclonal and polyclonal labelings. A simple approximation is that if 44% of injected fish have the transgene, then 44% of founders will harbor 2 transgene integrations. In order to correct for this bias, we multiplied the inferred number of precursors at the time of integration for each transmitting male by 1.44 to adjust for the presence of polyclonal founders in the dataset (Table 1). In this case, the median number of GCPs at the time of integration is 9.9 (Table 1) also during dome stage. Founders with different numbers of calculated GCPs at the time of transposon integration group into one of four developmental stages and are shown in Fig. 1D.

**Table 1:** Number of germ cell precursors (GCPs) inferred from EF1α>GFP transmission rates.

Founder Identity	GFP^**+**^ F1 Embryos	F1 Embryos Screened	% Transmission	Estimated GCPs at time of Integration^a^	Corrected GCPs at time of Integration^b^
17	75	277	0.271	1.8	2.7
44	70	268	0.261	1.9	2.8
31	9	35	0.257	1.9	2.8
8	76	401	0.190	2.6	3.8
42	133	755	0.176	2.8	4.1
18	72	445	0.162	3.1	4.5
43	163	1013	0.161	3.1	4.5
6	34	222	0.153	3.3	4.7
13	31	210	0.148	3.4	4.9
9	5	35	0.143	3.5	5.0
32	10	70	0.143	3.5	5.0
34	63	456	0.138	3.6	5.2
7	41	312	0.131	3.8	5.5
37	147	1152	0.128	3.9	5.6
14	147	1212	0.121	4.1	5.9
39	95	817	0.116	4.3	6.2
20	5	46	0.109	4.6	6.6
4	5	52	0.096	5.2	7.5
30	14	150	0.093	5.4	7.7
22	46	582	0.079	6.3	9.1
15	34	461	0.074	6.8	9.8
3	4	55	0.073	6.9	9.9
16	39	607	0.064	7.8	11.2
40	41	665	0.062	8.1	11.7
27	39	641	0.061	8.2	11.8
36	18	304	0.059	8.4	12.2
10	24	445	0.054	9.3	13.4
1	5	98	0.051	9.8	14.1
45	54	1074	0.050	9.9	14.3
12	14	289	0.048	10.3	14.9
35	24	499	0.048	10.4	15.0
23	55	1166	0.047	10.6	15.3
5	2	45	0.044	11.3	16.2
25	10	230	0.043	11.5	16.6
38	60	1507	0.040	12.6	18.1
21	28	813	0.034	14.5	20.9
26	17	556	0.031	16.4	23.5
11	13	516	0.025	19.8	28.6
41	24	958	0.025	20.0	28.7
24	2	100	0.020	25.0	36.0
19	10	515	0.019	25.8	37.1
29	7	501	0.014	35.8	51.5
33	9	862	0.010	47.9	69.0
28	1	250	0.004	125.0	180.0

a Estimated GCPs at integration = 1/(2×% transmission).

b Corrected GCPs at integration = 1.44×estimated GCPs.

Significantly, we find that integrations mostly occur after the midblastula transition, which confirms that Tol2 transposition generates sufficiently small clones for a variety of developmental analyses. Regardless of our treatment of the dataset (unadjusted or adjusted for polyclonality), we observe that the median # of GCPs at integration is between ∼7–10, which corresponds to the number of GCPs present at the 4,000-cell dome stage. Furthermore, we find that integration occurs over a relatively broad window of time during early development, suggesting we should be able to label melanocyte lineages at various stages of fate restriction.

### Generating rare melanocyte clones

**Figure 2:**
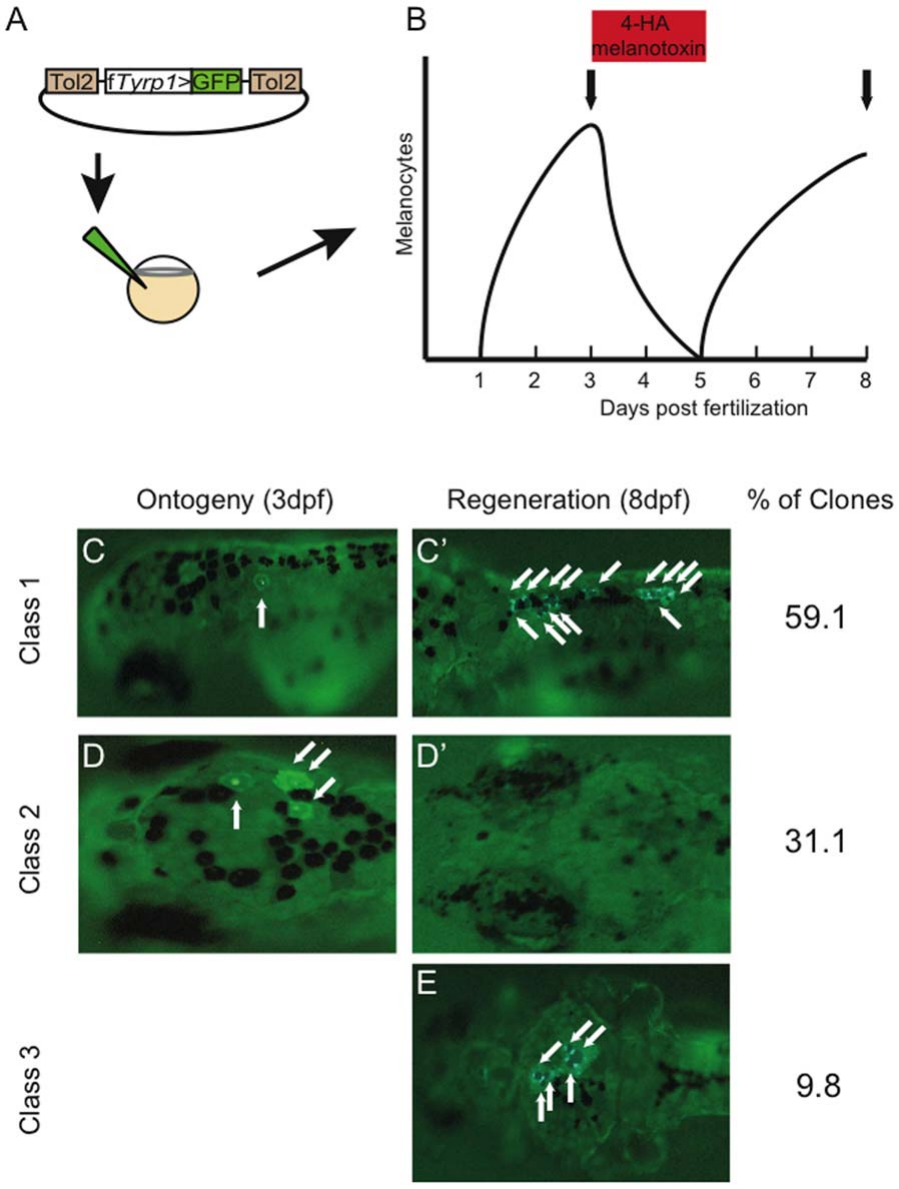
Three classes of melanocyte clones are revealed from clonal analysis. (A) Plasmid construct containing the melanocyte specific Takifugu rubripes *Tyrosinase related protein 1* promoter driving GFP (f*Tyrp1*>GFP) and flanked by Tol2 transposon arms was co-injected with transposase mRNA into 1–2 cell embryos. (B) Experimental protocol used for clonal analysis of melanocyte lineages. Following transposon injection, ontogenetic melanocytes are allowed to develop normally through 3 dpf, and then screened for GFP+ melanocytes. 4-HA treatment between 3 and 5 dpf was then used to ablate melanocytes. Drug was then washed out to allow melanocyte regeneration from MSCs. At 8 dpf fish are again screened for the presence of GFP in regeneration melanocytes. Vertical arrows indicate time of scoring for labeled melanocytes. (C–E) Representative pictures of fish showing ontogenetic melanocytes prior to ablation (C and D) and regeneration melanocytes following ablation and regeneration (C' and E). Note that the same fish is shown in C and C' as well as D and D'. Arrows point to GFP expressing ontogenetic and regeneration melanocytes. Fish with GFP labeled melanocytes can be divided in 3 clone classes: Class 1, with both ontogenetic and regeneration melanocytes labeled, indicating integration in a bipotent melanogenic precursor; Class 2, with only ontogenetic melanocytes labeled, indicating integration in a restricted direct-developing precursor; and Class 3, with only regeneration melanocytes labeled, indicating integration in a restricted MSC precursor.

To mark melanocyte lineages a construct containing the f*Tyrp1* promoter driving GFP flanked by the Tol2 transposable element arms was used (Figure 2A) [26], [29]. TYRP1 encodes an enzyme essential for synthesizing melanin, the black pigment that gives melanocytes their distinctive color and is uniquely expressed in melanocytes and the retinal pigment epithelium. Moreover, numerous stable transgenic lines bearing this transposon have been generated that express GFP in all melanocytes (not shown). Additionally, while lineages that develop into other tissues may have integrated the transposon construct, only differentiated melanocytes (and RPE) express the GFP reporter, making this transposon appropriate for lineage analysis of melanocyte development.

In total, 2715 fish were screened from eight separate days of injections. The amount of plasmid DNA and transposase mRNA injected into each fish was optimized to generate rarer clones than observed for generating Xef1α>GFP transgenics. The rate of producing melanocyte clones varied from day to day, ranging between 2.5% and 14.7%. While the majority of fish (93.8%) showed no evidence of labeled direct-developing melanocytes, 6.2% of injected fish were found to have at least one GFP+ ontogenetic melanocyte. This low rate of generating melanocyte clones suggests that a majority of fish carried single integration events into melanocyte precursors.

### Classifying clones by labeled melanocyte lineages

Fish were screened at 3 dpf for GFP expressing ontogenetic melanocytes (Figure 2B). The number and location of all labeled cells were noted to determine the clone size (number of melanocytes) and dispersion of the clone along the A–P axis. Treatment with 4-HA was then used to ablate the direct-developing melanocytes. Drug was then removed at 5 dpf to permit the melanocyte stem cell population to regenerate larval melanocytes. At 8 dpf, all fish were re-screened for the presence of GFP-labeled stem cell-derived melanocytes, again noting the location and number of regenerated cells. 15 fish with ontogenetic clones died prior to the end of regeneration and were not included in this analysis.

Approximately two-thirds (97/148) of fish with ontogenetic melanocyte clones (Fig. 2C) also regenerated labeled melanocytes (Figure 2C'), indicating a single neural crest precursor gave rise to both direct-developing melanocytes and the MSC. The other one-third (51/148) of ontogenetic clones (Fig. 2D) failed to regenerate a GFP labeled melanocyte (Fig. 2D'), suggesting integration in a melanocyte lineage that only produces direct developing melanocytes without segregating a related MSC. In 16 fish that were negative for GFP+ ontogenetic melanocytes, we found one or more labeled melanocytes following regeneration (Figure 2E) indicating integration of the transposon in a melanocyte precursor that only gave rise to a MSC.

This data allows us to place all fish that integrated the f*Tyrp1*>GFP transposon in a melanocyte precursor into three informative classes (Figure 2C–E & Supplemental Table S1). Class 1 clones were the largest class (59.1%) and produced both labeled direct developing melanocytes and MSCs capable of regenerating labeled melanocytes after ablation. Class 2 clones (31.1%) were less abundant and only contained direct-developing melanocytes. Class 3 clones (9.8%) contained no labeled direct developing melanocytes but regenerated labeled melanocytes from MSCs. Therefore we concluded that the majority of direct-developing melanocytes and melanocyte stem cells are derived from a common neural crest precursor in the early developing embryo.

**Figure 3:**
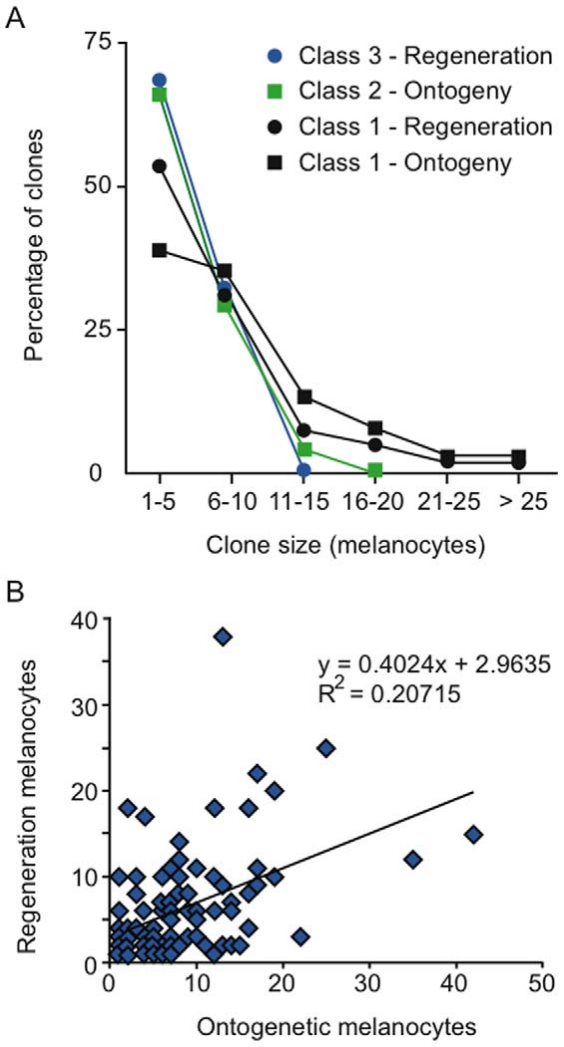
Clone size distributions. (A) Percentage of clones within each clone class relative to the size of the clone. (B) Number of ontogenetic melanocytes plotted against the number of regeneration melanocytes for each class 1 clone.

Distribution of clone sizes by class is shown in Fig. 3A, demonstrating the larger clone sizes apparent in class 1 clones relative to class 2 and class 3 restricted clones. Fig. 3B plots the size of ontogenetic melanocytes against the size of the regeneration melanocytes for each of the class 1 clones. Note that the number of regeneration melanocytes tends to increase with the number of ontogenetic melanocytes. Although this correlation is weak (R^2^ = 0.20715) it raises the possibility for cell division and clonal expansion prior to fate segregation in some integrants.

### Clone size and dispersion by class

The majority of clones (Class 1) indicate that melanogenic precursors commonly give rise to both direct-developing melanocytes and MSC(s). We explored the possibility that class 1 clones represent earlier integration events, occurring prior to the shield stage, while class 2 and class 3 clones represent later integration events that occur after segregation of the two cell fates from a shared precursor, perhaps after the shield stage. To test this hypothesis, we considered both the total number of melanocytes generated in these clone classes as well as the dispersion of labeled melanocytes within the fish. The earlier a neural crest precursor is labeled, the greater the opportunity for it to undergo cell divisions and generate multiple intermediate cells that could differentiate into direct developing melanocytes or MSCs. Additionally, from the shield stage to the end of gastrulation, cells undergo dramatic rearrangements in which epiboly, convergence, and extension disperse the clone along the A–P axis [30]. Cells that undergo earlier integration events are predicted to experience more of the spreading effects of gastrulation and should therefore be dispersed over more of the A–P axis of the resultant embryo than clones produced by later integration events.

To determine whether these class 1, 2, and 3 clones could represent different time points of integration, we analyzed the size and dispersion of each, the results of which we show in Table 2. Class 1 clones generate on average 8.2 ontogenetic melanocytes and 6.3 regenerated melanocytes from stem cells. These melanocytes are dispersed over extensive distances along the A–P axis of the fish, with direct developing melanocytes spanning an average of 11.9 somites per clone, and stem cell-derived melanocytes spanning an average of 9.0 somites. Consistent with the model that class 2 and class 3 clones represent later integration events, class 2 direct-developing clones are smaller in size (4.3 melanocytes, p<0.0005) and spread over a smaller portion of the A–P axis (7.2 somites, p<0.001) than the direct-developing melanocytes of class 1 clones. Similarly, class 3 MSC-only clones are smaller than the MSC-derived melanocytes of class 1 clones, producing an average of 4.3 melanocytes (p<0.025) and spread over an average of 4.8 somites (p<0.05). This data supports the hypothesis that class 2 and class 3 clones are predominantly due to late integration events occurring after segregation of the direct-developing and stem cell fates from a single melanogenic neural crest cell precursor.

**Table 2:** Clone properties of observed melanocyte classes.

Clone Class	N	%	Clone Size (Number of Melanocytes)	Clone Dispersion (Somites)	% Bilateral
1 Ontogeny	97	59.1	8.2+/−6.9^a^	11.9+/−9.7^c^	79 (67/85)
1 Regeneration	97	59.1	6.3+/−6.1^b^	9.0+/−9.2^d^	60 (49/82)
2 Ontogeny	51	31.1	4.3+/−3.1^a^	7.2+/−6.4^c^	74 (31/42)
3 Regeneration	16	9.8	4.3+/−2.7^b^	4.8+/−8.2^d^	79 (11/14)

Two-sample t tests were used to compare differences in clone size and clone dispersion between class 1 and class 2 ontogeny clones (^a,c^) and class 1 and class 3 regeneration clones (^b,d^). P values for comparisons as follows:

a p value<0.0005.

b p-value<0.025.

c p-value<0.001.

d p-value<0.05.

Two additional properties that were explored with this set of melanocyte clones were whether melanocyte lineages later mix with each other and whether lineages were restricted to one side of the midline. The majority of clones in the dataset clearly demonstrated mixing of melanocyte lineages is commonplace in zebrafish development, as unlabeled melanocytes regularly developed in between two clonally related GFP labeled melanocytes (Fig. 2C'). All clones with at least two melanocytes were examined to determine if the clone was restricted to one side of the midline or bilaterally expressed. Although 29.1% (65/223) of clones showed clear restriction to the left or right side of the larvae, the vast majority (70.9%) had bilaterally distributed melanocytes, suggesting that after convergence melanocyte precursors can cross the midline.

### Number of melanocyte lineage precursors

**Figure 4:**
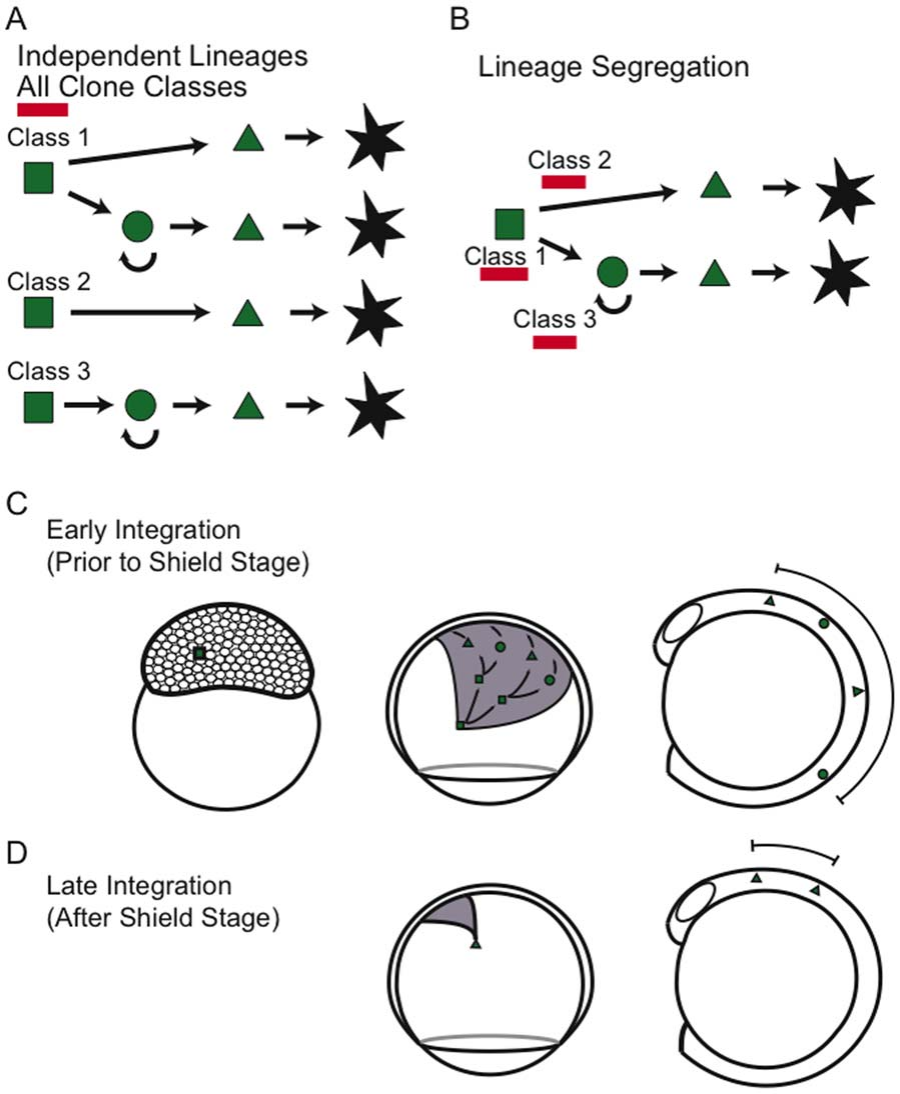
Alternative lineage models to explain clone classes. (A) Clone classes indicate independent lineages during the period of transposon integration or (B) clone classes represent different times of integration, either before (Class 1) or after (Class 2 and 3) segregation of fates from a common precursor. Red bars indicate time of integration in indicated clone class. Squares represent neural crest precursors, circles represent melanocyte stem cells, triangles represent melanoblasts or transient amplifying cells, and stars represent differentiated melanocytes. (C,D) Predictions for the effect of different times of transposon integration into a melanocyte precursor on clone size and clone dispersion over the A–P axis, using the model depicted in (B).

This dataset of rare melanocyte clones presents the opportunity to estimate the number of melanogenic precursors present in the early developing embryo. We will first consider the number of precursors using the model that class 1, 2 and 3 lineages are independent (Fig. 4A). In this case we solve that there are 40 class 1 precursors, 21 class 2 precursors, and 31 class 3 precursors (see Materials and Methods), for an average of 92 melanogenic precursors during the period of integration (on average at the dome stage).

Alternatively, we consider the model whereby class 2 and class 3 clones segregate from class 1 precursors sometime after the shield stage (Fig. 4B). Given that the typical 3 dpf larva has 420 differentiated melanocytes and the average ontogenetic clone size for all 3 dpf class 1 clones is 8.2 melanocytes, we estimate approximately 51 (420/8.2) separate bipotent precursor cells are present at the dome stage (the average time of integration). We can solve for the same precursors using the MSC-derived melanocytes in class 1 clones. Since the larvae regenerates 380 melanocytes, and the average class 1 regenerate clone is 6.3 melanocytes, this alternative calculation suggests 60 (380/6.3) bipotent precursors, in close agreement with the 51 precursors determined by using ontogenetic class 1 melanocytes.

We can use the same reasoning to estimate the number of targets present for labeling after segregation of the direct-developing and MSC fates. Thus, our finding that class 2 clones generate on average 4.3 melanocytes suggests approximately 98 direct-development restricted melanocyte precursors (420/4.3) are responsible for producing direct-developing melanocytes in the embryo. A similar result holds for the class 3 (regeneration only) clones, which produce an average of 4.3 melanocytes per clone. Since only ∼380 melanocytes regenerate in the larvae, we calculate that approximately 88 MSC restricted precursors (380/4.3) can be found in the embryo at times of labeling, after segregation from the bipotent precursor.

The foregoing analysis estimated the number of lineages at the time of labeling that generated MSCs. We can also put boundaries on the number of MSCs in the larval fish at the time it is challenged to regenerate. For this analysis we will use the second model (Fig. 4B). The minimal number of MSCs is the number of MSC-restricted lineages, which we calculate to be approximately 90. If MSCs produced regeneration melanocytes on a 1∶1 basis, then a single asymmetric cell division would be required to renew the MSC and produce a melanoblast for terminal differentiation, and sets the maximal number of MSCs at 380. Yang and Johnson [25] used BrdU labeling to demonstrate at least 2, and possibly 3, cell divisions occur between the recruitment of the MSC to regenerate melanocytes and the subsequent differentiation of new melanocytes. This reduces the maximal number of MSCs to 190. Together, these data suggest a range of 90 to 190 MSCs in the larval zebrafish. Conservatively, each lineage produces a single MSC that is recruitable at larval stages, while maximally each lineage produces ∼2 such MSCs.

## Discussion

### Tol2 transposon injection in 1–2 cell stage embryos results in transposition after midblastula transition

The time of integration of the Tol2 transposon has remained undefined since its inception as a technique for generating transgenics in the zebrafish [31]. The slow and deliberate expansion of the *vasa* expressing germ cell precursors over the first day of development allows us to estimate the timing of integration. Using germline transmission rates of a ubiquitous Xef1α>GFP reporter expressed on a Tol2 transposon from injected F0 founders to their offspring, we have shown that the average time of integration is approximately the dome stage (4.3 hpf), with the majority of integrations occurring from the midblastula transition to shortly after shield stage. Consequently the Tol2 transposon is a useful means of labeling clones during the early embryonic stages when germ layers and cell type specific lineages are being established. Integration may occasionally occur both early (2-cell) or extremely late (24 hpf). However, these times of integration are clearly at the tails of the distribution curve and represent a minority of the data in this type of analysis. We never see injected embryos that show GFP expression in the majority of cells when using a ubiquitous promoter such as Xef1α. Thus, it is unlikely that integration can ever occur in the 2-cell embryo as suggested by transmission rates (Founders 17, 44, and 31, Table 1). These founders may instead be the result of multiple integration events at later stages, when there are 4 germline precursors, or reflect unequal contributions of germline precursors to the gonad.

Our finding that most transposon integrations occur between the midblastula transition and shield stages, after somatic lineages have segregated away from the germline, is informative when considering the construction of transgenic lines. Screening and identification of interesting expression patterns in injected F0 founders is not a good predictor that the transposon will be transmitted to F1 progeny. With the use of a specific, non-ubiquitous promoter driving reporter expression, screening of both GFP+ and GFP− founders from an injected clutch is necessary, as both are equally likely to harbor the transposon in the germline. It also explains the finding from enhancer trap screens (Woolls, M., McMahon, R., and Johnson, S., not shown) that expression of reporters in founders does not correlate to expression in transmitted traps, since they arise from different chromosomal integration sites.

Although we have not explored why integration events occur late, it is useful to briefly consider the delay in Tol2 transposition. One salient feature of our analysis is that the onset of transposition is approximately coincident with the onset of the midblastula transition (at 1000 cell stage). This stage marks several events, including the acquisition of cell cycle control [32], [33], changes in chromatin [34], and global changes in transcription and translation [35]. Each of these has the potential to account for decreased transposition at early stages, and increased transposition after the midblastula transition. For instance, the possibility that the capped mRNA that is co-injected with the transposon is not available for translation until the midblastula transition could be tested by injecting in vitro translated transposase, or by adding 3′UTR of cyclin or c-mos genes to the transposase transcript, that allows regulated polyadenylation of the chimeric transcripts and promotes increased translation in the pre-midblastula transition embryo [36].

### Most direct-developing and MSC-derived melanocytes develop from a common neural crest precursor

Previous work from ours and other labs suggested that two different lineages of melanocytes contribute to the embryonic and larval melanocyte pattern: one that develops directly without an MSC intermediate, and a second population that develops from MSCs [1], [2]. These different populations develop at different times. The direct developing melanocytes arise before 3 dpf, while melanocytes that develop after 3 dpf, or during regeneration following melanocyte ablation develop from an *erbb3b*-dependent MSC [3]. We were interested in whether these two lineages were related through an earlier precursor during embryogenesis. To explore this, we generated clones using Tol2 with a melanocyte specific reporter, f*Tyrp1*>GFP. We combined this with sequential developmental assays, assessing first the contribution of clones to direct developing melanocytes, and subsequently to MSC-derived regeneration melanocytes. This analysis showed that the majority (59.1%) of melanogenic precursors marked by the transposon give rise to both direct developing melanocytes and MSCs.

We were also interested in whether clones that were restricted to direct developing melanocytes (class 2) or MSCs (class 3) were the result of later labeling events, following segregation of these fates from a precursor capable of generating both lineages, such as that labeled in class 1 clones. We reasoned that we could use the relative dispersion of clones along the A–P axis due to morphogenetic movements of gastrulation, including epiboly, convergence, and extension, to test whether class 1 clones were the result of earlier integration events than the class 2 and class 3 clones. Our analysis showed that not only did the class 1 clones generate more melanocytes of both the direct-developing type and the MSC-derived type than in the type restricted clones, but also that these melanocytes were dispersed over a greater extent of the A–P axis than the melanocytes of the restricted clones (Figure 4C). These clones suggest that direct-developing melanocyte-only and MSC-only clones are the result of late integration events following segregation from a bipotent precursor (Fig. 4D). Together with the finding that the majority (59.1%) of integration events occurred in a bipotent precursor, these results support a model that most or all direct-developing and MSC lineages are derived from a common melanogenic precursor in the early gastrula (Fig. 4B, C).

While we favor the model that class 2 and class 3 clones are a result of late integration events, alternative hypotheses exist that could explain the data for restricted lineages. Failure to recruit a MSC to regenerate would make a class 1 clone appear as a class 2 clone. Double regeneration experiments (O'Reilly-Pol and Johnson, unpublished data) using similar clonal analysis suggests that the vast majority of, but not all, MSCs are recruited using our protocol. Our finding that 35% of clones giving direct-developing melanocytes fail to regenerate is far greater than the fraction of unrecruited MSCs observed during double regeneration experiments. Additionally, although we suggest that class 2 and class 3 lineages are derived from a class 1 lineage, it still remains possible that embryos simultaneously produce a limited number of direct-developing or MSC-only lineages that are not related through a common precursor (Figure 4A). Based on the proportion of clones generated in this dataset, these class 2 and class 3 clones would still represent a minority of melanocyte lineages in the zebrafish. We also note that our analysis does not exclude the possibility of other neural crest derivatives, such as other pigment cells or other stem cells, segregating from these lineages.

### Timing of fate segregation from a bipotent melanogenic precursor

Combining the axial dispersion of our melanocyte clone classes with the timing of transposon integration, we can begin to speculate when the single precursor that results in class 1 clones segregates into two separate fates, thereby giving class 2 and class 3 clones. Taking into consideration the corrections made for polyclonal contributions to the Xef1α>GFP transposon dataset, ∼73% (32/44) of the transposition events have occurred prior to the shield stage. That class 1 clones make up 58% of our transposition events may suggest that the segregation of the bipotent precursor into the direct-developing and stem cell lineages occurs after the shield stage. While the timing of this segregation may seem early, segregation of the primitive hematopoietic lineages, specifically those that are unipotent (macrophages only) from multipotent (erythrocytes, neutrophils, thrombocytes) has been shown to occur by 6 hpf [9]. Further supporting the timing of segregation is the fact that class 2 and class 3 clones show modest, yet notable, dispersion along the A–P axis (7.2 and 4.8 somites, respectively). If segregation of these melanocyte lineages was occurring much later in development, for instance following convergence and extension, then a class 2 or 3 clone would be predicted to span a relatively narrow region (1 or 2 somites) since migration along the AP axis following emergence from the neural crest is generally minimal [37].

It is useful to begin to consider how the melanocyte lineages observed in zebrafish compare to those observed in the mouse. As is true of all vertebrates, mouse melanocytes are neural crest derivatives that begin migration away from the neural tube as melanoblasts, and can be visualized with the DCT-LacZ reporter construct beginning at E10.5 [38]. Over the next four days, melanoblasts populate the entire ventrum of the mouse, beginning in the head and cervical region and continuing in a wavelike manner toward the posterior. By E14.5, melanoblasts begin migrating into hair follicles, at which point a subset of cells terminally differentiate to form the melanocytes of the first hair cycle, while a smaller portion are converted into MSCs that reside in the bulge region of the hair follicle [39]. Based on our results, segregation of fates from the bipotent melanogenic neural crest precursor occurs at a considerably earlier stage in development in zebrafish (pre neural crest) than is seen in the mouse (post neural crest).

This apparent heterochrony of MSC segregation from a precursor between simple vertebrates and mammals is notable. One possibility is that the evolution of the hair follicle as the site for MSCs led to a delay of segregation of the MSC fate until this niche was present to accept them. This may argue that the niche for the zebrafish MSC is available at early stages, perhaps immediately after migration from the neural crest.

### Properties of melanogenic precursor pools used to establish pigment pattern

Two fundamental issues of pigment pattern establishment can be addressed using clonal analysis presented here. Namely, the number of melanogenic precursors that exist in the developing embryo, and the behavior of the melanogenic precursors in relation to one another. First, the size of the melanogenic precursor pool responsible for establishing pigment pattern was initially explored in the mouse by studying aggregation chimeras formed by mixing 8 cell morulae from differently pigmented lines and analyzing the resultant pigment patterns in the adult [40]. Mintz [40] noted 17 transverse bands of coloration which fail to cross the midline, leading her to propose 34 melanogenic precursors in the neural crest. More recent studies of aggregation chimeras expressing the *DCT*-LacZ reporter (discussed above) have indicated a greater number of melanogenic precursors than was first described [41]. In particular the authors noted that nearly all chimeras exhibited *DCT*-LacZ positive clusters in the head and cervical region, suggesting a more substantial pool of melanogenic precursors intermixing and populating this region than was suggested from the earlier work. Our work suggests between 60 and 100 precursors for the embryonic pattern, depending on the model of fate segregation and timing of integration, consistent with the more recent mouse work suggesting many precursors.

Secondly, the behavior and interaction of multiple melanocyte precursors can also be investigated with clonal analysis. Mintz reported observing distinct boundaries between adjacent differently pigmented regions of the mouse, in which crisp borders delineated the two regions and indicated little intermixing of the pigmented descendents from the two presumptive precursors [40]. Again, use of the *DCT*-LacZ reporter in mouse embryos showed a different result, with a greater degree of intercalation of melanocyte lineages than previously reported [41]. We also observe extensive mixing of melanocyte lineages, as GFP+ labeled melanocytes are regularly interspersed with unlabeled melanocytes both during ontogeny and regeneration.

Finally, the reports of both Mintz and Wilkie suggest midline restriction of precursors and their progeny. In both adult pelts and in embryonic in situ analysis, clones on the right and left side of an embryo behave independently from one another and are restricted with respect to the midline [40], [41]. Interestingly, our data shows that both ontogenetic melanogenic precursors and MSC-restricted lineages are generally capable of producing differentiated daughter cells on both sides of the midline. Overall, zebrafish larvae do not show midline restriction in melanocyte clones, with only 29% of clones strictly limited to one side of the fish.

## Materials and Methods

### Fish stocks and husbandry

All animals were housed, reared, bred, and anesthetized in accordance with protocols approved by the Washington University Animal Studies Committee (Protocol 20080311). Fish stocks were housed and reared according to The Zebrafish Book [42]. We used the inbred AB line sjA to generate Xef1α>GFP lines for assessing time of integration, and *mlpha* ^j120^ mutants for clonal analysis in melanocytes [43]. *mlpha* ^j120^ is a viable mutation in *melanophilin*, which results in melanocytes with their melanosomes contracted to the center, allowing for easier identification of GFP expression in the cell periphery.

### Calculating the number of germ cell precursors (GCPs) at integration

The transmission rate of the Xef1α>GFP transposon from an F0 founder to its F1 progeny is proportional to the percentage of the founder's germline cells that integrated the transposon. Since gametes are haploid, the transmission rate must be doubled, leading to the equation:
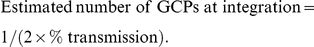
Since 44% of Xef1α>GFP injected fish were capable of transmitting the transposon to its progeny, and integration events are assumed to occur independently of one another, we estimate that 44% of F0 founders actually harbor two germline integrations. A multiplier of 1.44 was applied to all unadjusted GCP calculations to compensate for the fact that multiple integrations of a founder's germline will inflate the observed transmission rates and make integration times appear earlier in development.

### Generating melanocyte clones

Zebrafish embryos were produced by in vitro fertilization and injected at the 1–2 cell stage, as previously described [29], [44]. 1 µL of DNA plasmid (15 ng/µL) containing the f*Tyrp1*>GFP reporter flanked by Tol2 transposon arms [26] was combined with 1 µL of 15 ng/µL capped 5′ transposase mRNA, and 1 µL of 1% phenol red in a 15 µL total volume. Approximately 5 nL of solution was injected into each 1–2 cell embryo.

### Identifying and Analyzing Clones

Embryos were anesthetized in tricaine methanesulfonate and screened for GFP expression on an epifluorescent stereomicroscope. For embryos containing GFP+ melanocyte clones, the location of all labeled melanocytes was recorded with regard to both the A–P (somite) and D–V (stripe) axes. The head was subdivided into three sections, H1 to H3, where H1 included areas anterior to the midline of the eyes, H2 extended from the midline of the eyes to the anterior edge of the operculum, and H3 included the operculum and regions anterior to somite 1. Two-sample t tests were used to analyze differences in clone size and clone dispersion in different melanocyte clone classes.

### Drug-induced melanocyte ablation

At 3 dpf, following the initial screen for GFP-expressing direct-developing melanocytes, all fish were placed in a 4 µg/mL solution of 4-hydroxyanisole (4-HA, M1865-5, Sigma Aldrich). Fish with direct-developing melanocyte clones were individually treated with 4-HA and reared for the remainder of the experiment in 12-well cell culture plates. Fish without labeled direct-developing melanocytes were pooled in groups of 50 in Petri dishes and similarly treated with 4-HA to reveal MSC-derived melanocytes. Following 2 days of 4-HA treatment, melanocyte ablation was confirmed by the contraction and extrusion of these cells through the skin [25]. Loss of GFP expression was also used to confirm cell death of labeled direct-developing melanocytes. 4-HA was then washed out and fish returned to fresh water to allow regeneration of melanocytes. At 8 dpf, after 3 days of regeneration, all fish were re-screened for the presence of GFP-expressing stem cell-derived melanocytes.

### Solving for the number of melanogenic precursors

Two distinct models (See Figures 4A and B) illustrating the relationship of melanocyte lineages in the larval zebrafish can be utilized to solve for the number of melanogenic precursors at the time of integration. For our first model, this assumes that class 1, 2 and 3 melanocyte clones represent integrations into distinct, unrelated melanogenic precursors at the same time of development (Fig. 4A), and that the ratio of class 1 and class 2 clones reflects the ratio of their precursors. We solve for the approximate number of precursors of each clone class using total ontogenetic melanocyte counts at 3 dpf (420 melanocytes) and total regeneration melanocytes at 8 dpf (380 melanocytes) using the following 3 equations:

1. Class 1 precursors×8.2 melanocytes/precursor+Class 2 precursors×4.3 melanocytes/precursor = 420 ontogenetic melanocytes
2. Class 1 precursors×6.3 melanocytes/precursor+Class 3 precursors×4.3 melanocytes/precursor = 380 regeneration melanocytes
3. Class 1 precursors/class 2 precursors = 1.9

For the alternative model, that restricted lineages segregate from a bipotent precursor (Fig. 4B), we can solve for the number of bipotent precursors as the number of class 1 direct-developing or stem cell-derived melanocytes divided by their respective clone sizes. Similarly, the number of direct-developing restricted precursors at the time of integration is solved by dividing the number of ontogenetic melanocytes by the class 2 clone size, and the number of melanocyte stem cell restricted precursors is solved as the number of regeneration melanocytes divided by the class 3 clone size.

## Supporting Information

Table S1 Quantitative and qualitative description of transposon labeled melanocyte clones. The number of GFP-labeled ontogenetic (Class 1 and 2) and regeneration (Class 1 and 3) melanocytes counted in the assay is shown for each individual clone. Clone span details the dispersion of the labeled melanocytes along the A–P axis. The head was divided into three sections, H1 to H3 (described in materials and methods), with H1 being anterior and H3 being posterior and adjacent to somite 1. S stands for somite with 1 being most anterior and 31 being most posterior. Clone length is a quantitative measure of clone span, with each section (H or S) counted as 1 somite. Bilateral clones were defined as containing at least two labeled melanocytes on opposite sides of the midline.
(XLS)
